# Kinematic relationship between rotation of lumbar spine and hip joints during golf swing in professional golfers

**DOI:** 10.1186/s12938-015-0041-5

**Published:** 2015-05-14

**Authors:** Frederick Mun, Seung Woo Suh, Hyun-Joon Park, Ahnryul Choi

**Affiliations:** Department of Biological Sciences, Carnegie Mellon University, 5000 Forbes Avenue, Pittsburgh, PA 15213 USA; Department of Orthopedics, Scoliosis Research Institute, Korea University Guro Hospital, 148 Gurodongro, Guro, Seoul, 152-703 Republic of Korea; Department of Bio-Mechatronic Engineering, College of Biotechnology and Bioengineering, Sungkyunkwan University, 2066 Seoburo, Jangan, Suwon, Gyeonggi 440-746 Republic of Korea

**Keywords:** Golf, Lumbar spine, Hip, Rotational movement, Coupling

## Abstract

**Background:**

Understanding the kinematics of the lumbar spine and hip joints during a golf swing is a basic step for identifying swing-specific factors associated with low back pain. The objective of this study was to examine the kinematic relationship between rotational movement of the lumbar spine and hip joints during a golf swing.

**Methods:**

Fifteen professional golfers participated in this study with employment of six infrared cameras to record their golf swings. Anatomical reference system of the upper torso, pelvis and thigh segments, and the location of each hip and knee joint were defined by the protocols of the kinematic model of previous studies. Lumbar spine and hip joint rotational angle was calculated utilizing the Euler angle method. Cross-correlation and angle–angle plot was used to examine the degree of kinematic relationship between joints.

**Results:**

A fairly strong coupling relationship was shown between the lumbar spine and hip rotational movements with an average correlation of 0.81. Leading hip contribution to overall rotation was markedly high in the early stage of the downswing, while the lumbar spine contributed greater towards the end of the downswing; however, the relative contributions of the trailing hip and lumbar spine were nearly equal during the entire downswing.

**Conclusions:**

Most of the professional golfers participated in this study used a similar coordination strategy when moving their hips and lumbar spine during golf swings. The rotation of hips was observed to be more efficient in producing the overall rotation during the downswing when compared to the backswing. These results provide quantitative information to better understand the lumbar spine and hip joint kinematic characteristics of professional golfers. This study will have great potential to be used as a normal control data for the comparison with kinematic information among golfers with low back pain and for further investigation of golf swing-specific factors associated with injury.

## Background

Two of the most essential skills involved in golf, a sport of getting balls into a series of holes by hitting them with golf clubs, are directional accuracy and increased flying distance of the ball [1]. Most current golf lessons focus on the increased twisting of the trunk during the backswing (BS), since this motion stores the rotational energy that is released during downswing (DS), leading to higher ball speed and longer flight distance [2]. In addition, proper weight shifting during the golf swing is crucial for increasing the flight distance of the ball [3]. This weight transfer, controlled by the leading and trailing legs, has prominently different roles during the golf swing [4].

Golf is a high-risk sports in regards to the number of associated injuries [5]. The injury rate has shown a steady increase over the years, with low back pain (LBP) presenting as the most common injury, accounting for 25–36% of the overall reported injuries [6–8]. There are several speculated causes of LBP, such as excessive twisting of the lumbar spine and subsequent derotation when the player reaches the top of the BS, or hyperextension and excessive torque of the lumbar during the DS and follow-through [9]. However, the accurate etiology of LBP is still unknown [10, 11]. Therefore, identifying the risk factors related to LBP is necessary for the prevention of injuries and establishment of effective treatment strategies.

According to previous publications, LBP is strongly associated with altered mobility of the lumbar spine and hip joints [12]. The kinematics of the lumbar spine and hip joint can be influenced in various ways by injuries and therapeutic intervention [13]. Lee and Wong [14] demonstrated a high coupling between movements of the lumbar spine and hip joints using cross-correlation analysis. Their study was focused on the coordination of movement in three anatomical planes. Specifically for the golf swing, Vad et al. [9] reported that history of LBP was associated with decreased lumbar extension and leading hip rotation, as determined using FABERE’s distance and finger-to-floor test. More recently, Murray et al. [15] found that a group of amateur golfers with LBP had a reduced medial range of motion of the hip joints compared to the control group. These findings imply that it is important to understand the kinematic relationships and coordination between the lumbar spine and hip rotation during a golf swing of the golfers with and without LBP. Ultimately, this will provide valuable insight for determining the golf swing-specific factors of LBP, and subsequently developing the therapeutic strategies for the prevention of LBP.

To the best of our knowledge, none of the previous studies specifically explored the kinematical relationship between the lumbar spine and hips during the golf swing. As a first step, the objective of this study was to examine the relationship between rotational movement of hips and the lumbar spine during the golf swings of professional golfers. It was hypothesized that there would be a high coupling relationship between rotational movement of the lumbar spine and each hip joint. Additionally, it was hypothesized that the characteristics of this relationship would differ greatly between the leading and trailing limbs, and between the BS and DS phases of a golf swing.

## Methods

### Subjects and apparatus

Fifteen professional golfers with no past history of musculoskeletal injury participated in this study. All participants were registered in the Korea Professional Golf Association, and were right-handed. This study was approved by the local ethics committee, and written informed consent was obtained from every participants before all experiments. The physical characteristics of the participants are shown in Table 1.

**Table 1:** Subject characteristics

	Professional golfers (N = 15)
Sex	Males
Age (years)	31.0 ± 6.0
Height (cm)	175.2 ± 8.7
Weight (kg)	72.3 ± 10.0
Handicap (strokes)	<0

**Figure 1:**
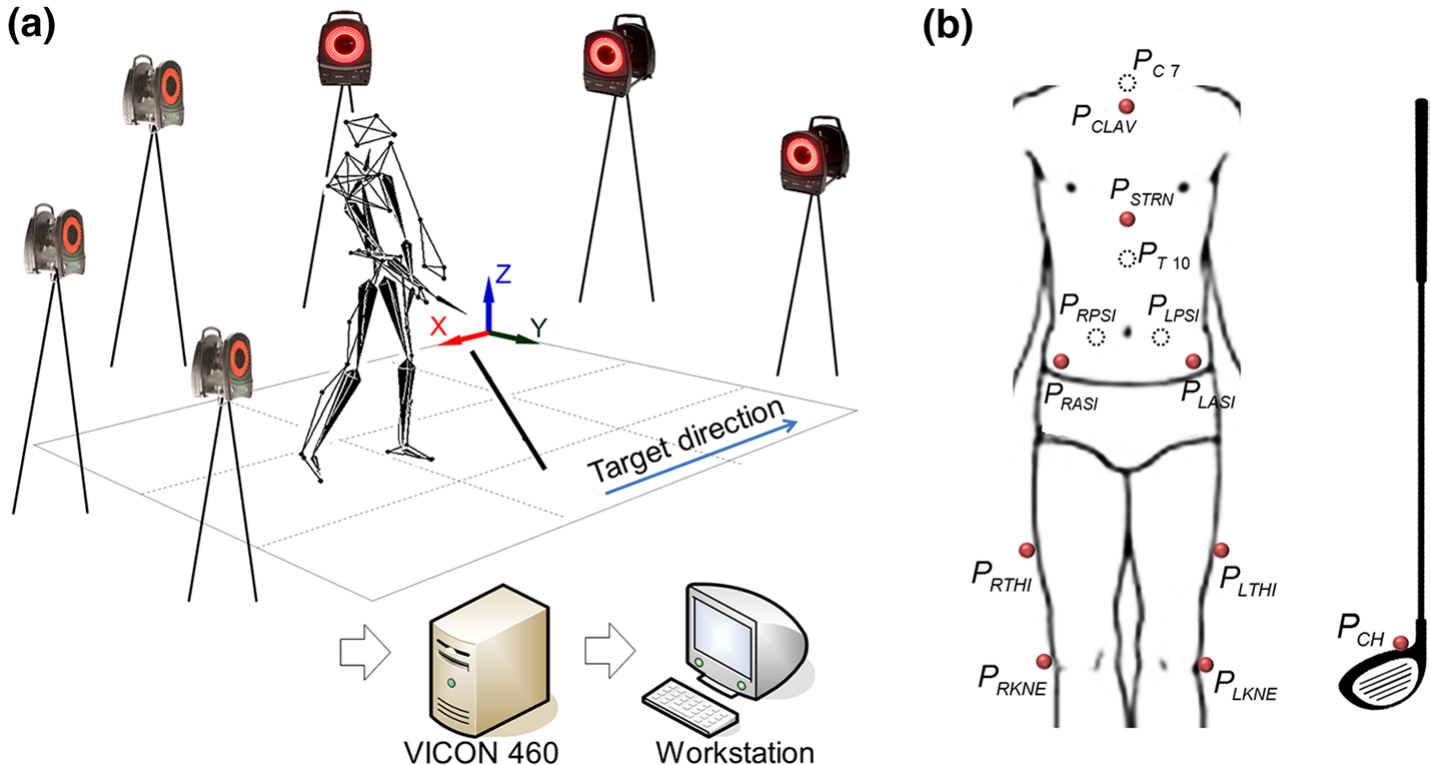
Golf swing analysis system: six infrared cameras, VICON 460 system and workstation software for interface between each device (**a**), and the location of markers attached on the body surface and club (**b**).

In order to set-up a similar environment as an actual indoor driving range, a 5 m × 5 m square net was created, and a swing mat was placed across the tee and both feet. Six infrared cameras (VICON460, Oxford Metrics, Oxford, UK) were used as measurement devices, with the capturing speed set to 120 Hz. The cameras were calibrated for reconstruction of the three-dimensional coordinates of the markers, and the origin of the global reference system was located in the left rear region. Based on the setup position of the participants, the X-, Y-, and Z-axes were in the left/right, forward/backward, and vertical directions, respectively. Figure 1a shows the overall experimental system.

### Experimental procedures

All golfers wore only a short sports bottom with no top. A total of twelve optical markers were attached on the skin of upper torso, pelvis, left thigh, and right thigh segments. Four markers were located on the suprasternal notch (P_CLAV_), xiphoid process (P_STRN_), the spinous process of C7 (P_C7_) and T10 (P_T10_). The other eight markers were placed as follows: left and right anterior superior iliac spine (P_LASI_ and P_RASI_), left and right posterior superior iliac spines (P_LPSI_ and P_RPSI_), left and right surface of the thigh segment (P_LTHI_ and P_RTHI_), and left and right femoral condyle (P_LKNE_ and P_RKNE_). In order to detect the impact stage, one additional marker and reflective tape were placed on the clubhead (P_CH_) and the ball, respectively. Figure 1b demonstrated the locations of the attached markers. The participants then warmed up with large dynamic movements and static stretches [16], and each subject was allowed to adapt to the laboratory environment with a practice swing [17]. Three swings were repeated per subject, and ensemble average data were used in the analysis. Data analysis was limited to the BS and DS phases, and the three events (address, backswing top, and impact) that defined the beginning and end of each phase. Address was defined as the set posture just before the initial club movement. Backswing top was when the club head had maximal rotation, and impact was the moment when the club head made contact with the ball [18]. These phases and events were distinguished by the trajectory obtained from the marker attached to the clubhead. Each of the raw 3D marker trajectories was extracted using SB-Clinic software (SWINGBANK Ltd, Republic of Korea) and filtered using a zero leg, 4th order low-pass Butterworth filter [19, 20]. The cutoff frequency was set to 10 Hz, as determined by a similar previous study [21].

### Lumbar spine and hip joint angular kinematics

**Figure 2:**
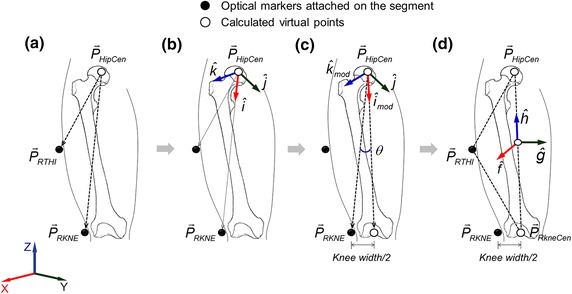
Procedures of thigh kinematics through **a** to **d**: development of anatomical reference system of thigh segments and determination of knee joint location.

The anatomical reference system of upper torso and pelvis segments, and the location of each hip joint were defined by the methods used in previous studies [22–25]. For a thigh anatomical reference system, a plane was constructed based on the locations of the hip joint rotation center (P_HipCen_), thigh (P_RTHI_) and femoral condyle marker (P_RKNE_) (Figure 2a, b). In consideration of the knee width of each participant, the knee joint rotation center was calculated by the medial direction offset parallel with the plane. The calculation of the knee joint center was carried out as following Eqs. (1)–(4):

1 $$\hat{i} = \frac{{\vec{P}_{RKNE} - \vec{P}_{HipCen} }}{{\left\| {\vec{P}_{RKNE} - \vec{P}_{HipCen} } \right\|}},\quad \hat{j} = \frac{{\left( {\vec{P}_{RTHI} - \vec{P}_{HipCen} } \right) \times \left( {\vec{P}_{RKNE} - \vec{P}_{HipCen} } \right)}}{{\left\| {\left( {\vec{P}_{RTHI} - \vec{P}_{HipCen} } \right) \times \left( {\vec{P}_{RKNE} - \vec{P}_{HipCen} } \right)} \right\|}},\quad \hat{k} = \hat{i} \, \times \, \hat{j}$$

2 $${}^{G}\left[ R \right]^{A} = \left[ {\begin{array}{*{20}c} {a_{11} } & {a_{12} } & {a_{13} } \\ {a_{21} } & {a_{22} } & {a_{23} } \\ {a_{31} } & {a_{32} } & {a_{33} } \\ \end{array} } \right] = \left[ {\hat{i}\quad \hat{j}\quad \hat{k}} \right]$$

3 $${}^{G}\left[ R \right]^{A}_{\bmod } = {}^{G}\left[ R \right]^{A} \times Rot(y,\;\theta )$$

4 $$\vec{P}_{RKneCen} = \vec{P}_{HipCen} + {}^{G}\left[ R \right]^{A}_{\bmod } \times {}^{A}\left( {\vec{P}_{RKNE} - \vec{P}_{HipCen} } \right)$$

where *θ* refers to the angle between the line connecting the center of the hip joint and the femoral condyle marker, and the line connecting the center of the hip joint and the center of the virtual knee joint (Figure 2c). The thigh anatomical reference system was built based on the locations of the femoral epicondyle marker, hip and knee joint center (Figure 2d). The z-axis was determined as a unit vector from the knee joint to the center of the hip joint, and the x-axis was set as a vertical direction of the plane consisting of these three coordinates. The y-axis was calculated by a cross product of the x- and z-axes.

The x, y and z-axes of the anatomical reference system of each segment were set as the anterior/posterior, medial/lateral and proximal/distal directions, respectively. Lumbar spine and hip joint rotational angle was calculated using the Euler angle method, which represents the difference of orientation of each segment.

### Data and statistical analysis

Cross-correlation is similar to the convolution operation of two signals, and is used for the similarity analysis of two continuous function patterns [26]. The maximum absolute value of the correlation coefficient was used to evaluate the inter-joint coordination between the continuous signals of the lumbar spine and hips. The ratios of the maximum movements of the leading and trailing hips to that of the lumbar spine during the BS and DS phases were examined to identify the relative contribution of each part at the backswing top and impact events. In addition, angle–angle plots were used to reveal how the rotational movements of each joints arrived at the backswing top and impact events. In order to quantify the contribution of rotational movement of the hip joints during the BS and DS, the slopes (hip rotational angle/lumbar spine rotational angle) of a line connecting the address to the backswing top point, and the impact to the backswing top point were calculated from the angle–angle plots.

The Shapiro–Wilk test was employed to evaluate data normality of all dependent variables. A paired *t* test was used for a comparative analysis to determine whether there were any differences between the BS and DS phase, and between the leading and trailing side. All statistics were processed using the SPSS statistical analysis program version 18.0.0 (SPSS Inc., Chicago, IL), and the significance level was set at p < 0.05.

## Results

**Figure 3:**
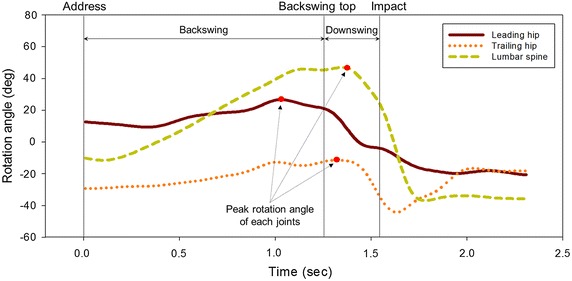
Ensemble angle of leading and trailing hip, and lumbar spine during the golf swing of a representative professional golfer. *Red points* represent the peak values of each joint angle. Positive degrees in the lumbar spine, leading hip, and trailing hip joints represent right axial rotation, external rotation, and internal rotation, respectively.

The ensemble angle profiles of the leading hip, trailing hip and lumbar spine during one golf swing of a representative golfer are illustrated in Figure 3. The rotational angle of each joint peaked at different times, but it could be seen that progression from the address to the backswing top involved clockwise rotation of the lumbar spine, external rotation of the leading hip and internal rotation of the trailing hip joint. During the DS phase, each joint then rotated in the opposite direction to return to a similar posture as the address position. Average peak values were 26.6° ± 11.2°, 4.4° ± 8.8°, and 45.5° ± 5.2° in the leading hip, trailing hip and lumbar spine joints of all golfers, respectively. All of the participants showed similar movement patterns.

The cross-correlation of the whole golf cycle represented a fairly high degree of relation between the lumbar spine and leading hip joint, and between the lumbar spine and trailing hip joint, with averages of r = 0.83 and 0.79, respectively (Table 2). In particular, the peak correlation coefficient between the lumbar spine and leading hip joint during the BS phase was similar to that between the lumbar spine and trailing hip joint, at r = 0.89 and 0.88, respectively. However, a small correlation coefficient (r = 0.65) between the lumbar spine and leading hip was observed during the DS phase, which suggested that the degree of association was weak compared to other phases, as well as to the other side (p < 0.01). In addition, the average time lags at peak rotation angle were 0.25 and 0.11 s for the lumbar spine versus leading or trailing hip, respectively. T tests results showed that the mean time lags were significantly different from zero (p < 0.01). Therefore, the leading and trailing hips generally preceded the lumbar spine in rotational movement during the golf swing. The rotation angle of the leading hip generally peaked earlier than that of the trailing hip (p < 0.01). It was concluded that the timing of the peak rotation angle represented the order of movement of leading hip, trailing hip and lumbar spine during the golf swing.

**Table 2:** Results of cross-correlation analysis of the rotational movement between the lumbar spine and hip joints

	Total swing	Backswing	Downswing
Lumbar spine and leading hip joint			
Max. correlation coefficient	0.83 ± 0.09	0.89 ± 0.16*	0.65 ± 0.13
Time lag at each peak value (s)	0.25 ± 0.12^§,£^	–	–
Lumbar spine and trailing hip joint			
Max. correlation coefficient	0.79 ± 0.10	0.88 ± 0.19	0.82 ± 0.18
Time lag at each peak value (s)	0.11 ± 0.13^§^	–	–

* p < 0.01 versus downswing, ^§^p < 0.01 versus zero time lag, ^£^ p < 0.01 versus lumbar spine and trailing hip joint.

**Figure 4:**
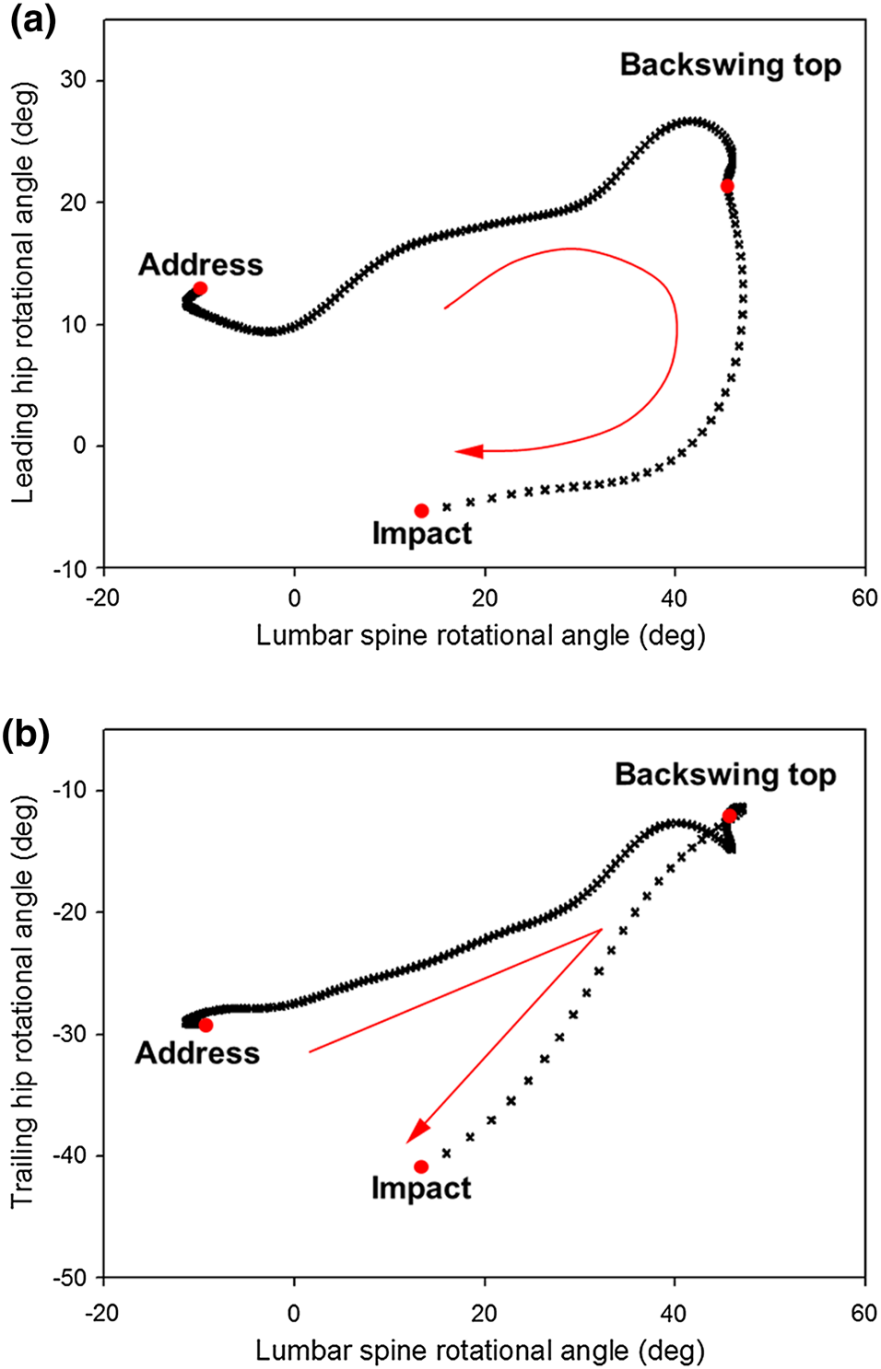
Angle–angle plots of rotational movement of lumbar spine versus the leading (**a**) and trailing hip (**b**) joints of a representative professional golfer. The major swing events (address, backswing top and impact) were shown as *red points*. Positive values of each axis indicate that lumbar spine rotated to the right, leading hip rotated externally, and trailing hip rotated internally.

Angle–angle plots of the rotational movements of the lumbar spine and each hip joint for a representative golfer are presented in Figure 4. The shapes of the curves were quite different when the angle graph of the leading hip versus lumbar spine was compared to that of the trailing hip versus lumbar spine. In the graph comparing leading hip versus lumbar spine (Figure 4a), the curve was a reverse ‘C’ pattern, indicating that the leading hip had a greater contribution to the initial DS phase of rotational movement, while lumbar spine rotation occurred predominantly near the impact position. However, the curve of the trailing hip versus lumbar spine graph exhibited a reverse ‘V’ shape, and it can be seen in the figure that the curve was almost a straight line during the DS phase. This suggested that the relative contributions of the trailing hip and lumbar spine rotation were near equal during the DS phase. Fourteen of the golfers displayed these patterns, while the remaining one was observed to have irregular-shaped curves.

**Figure 5:**
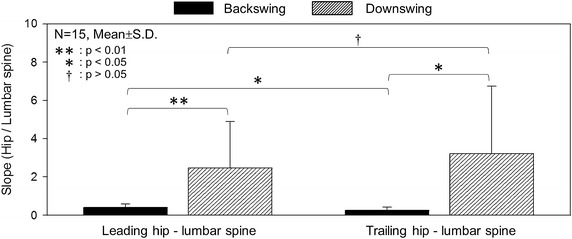
The average and standard deviations of the ratios of rotation of the lumbar spine and hip joints during the BS and DS phases. *Black filled squares* represent the BS phase, and *upward diagonal patterns* represent the DS phase.

The mean leading and trailing hips versus lumbar spine rotational angle ratios (the slope of the plot in Figure 3) were 0.40 ± 0.2 and 0.25 ± 0.2 for the BS phase, and 2.47 ± 2.4 and 3.21 ± 3.5 for the DS phase, respectively (Figure 5). The ratio of the DS was higher than that of the BS for both the leading and trailing hip, representing a statistically significant difference (p < 0.01). It was concluded that rotation movement in the DS phase was markedly achieved by the contribution of hip rotation.

## Discussion

Previous publications and case studies indicated a close association of hip joint mobility with LBP [27–29]. Specifically, significant differences between groups of professional golfers with and without a history of LBP were observed in lumbar extension, FABERE’s distance and hip internal rotation of the leading leg [9]. A group of amateur golfers with LBP displayed a significantly reduced active and passive medial rotation of the leading hip joint [15]. Such results demonstrated a strong relationship between mobility of the lumbar spine and hip joint. However, there have been limitations in understanding the accurate etiology and direct factors related to the association of LBP with the golf swing, because the kinematic relationship during the golf swing has yet to be studied. Time history patterns of the hips and lumbar spine kinematics can provide further insight into the process of movement and coordination of the joints during the golf swing from a biomechanical perspective.

Lee and Wong [14] examined the kinematic relationship between movements of the hip and lumbar spine during forward and backward bending, lateral bending and axial rotation. Similar contributions from the hips and lumbar spine were observed during the forward bending motion overall, but the lumbar spine was found to contribute more during the initial step of that movement. Lateral bending was primarily performed by the lumbar spine, while twisting motion was mainly accomplished by hip movements. However, a golf swing, as an intended movement to increase the speed of the ball at the impact position, is a complex and asymmetric series of movements involving three anatomical planes at once. In addition, the leading hip moves in a completely different fashion compared to the movement of the trailing hip, as suggested by the results obtained herein. Therefore, the concepts of previous studies would be insufficient for explaining golf swing mechanics in their entirety.

Although the anatomical axial rotation demonstrated comparable patterns in the lumbar spine, leading, and trailing hip joints (Figure 3), each peak value was observed at relatively different time points (Table 2). This indicates that each joint played different roles for twisting motion in a complete golf swing. The precedence of the peak rotation timing of both hip joints compared to the lumbar spine is closely related to the separation between the upper torso and pelvis rotation, which is defined as ‘X-factor’ or ‘X-factor stretch’. This is a swing strategy to increase a flight distance of the ball by maximizing the stored energy due to an increase of torso coiling [2]. Remarkable contribution of hip rotation in the DS phase than in the BS phase attributes pelvis-lead swing characteristics existed among the common skilled golfers. In particular, it could be suggested that the axial rotation of the leading hip affects twisting motion in the early DS phase of professional golfers (Figure 4a); therefore, the training for the proper rotation of leading hip allows to help joint coordination strategy between lumbar spine and leading hip joint.

The results of this study can be summarized as follows:

1. A fairly strong coupling relationship was displayed between the lumbar spine and hip rotational movements, with an average correlation of 0.81. This indicated that most of the professional golfers used a similar coordination strategy in moving their hips and lumbar spine to complete their swings.
2. During the DS phase, the leading hip was markedly rotated in the early stage, while the lumbar spine had a greater contribution near the impact position. However, the relative contributions of the trailing hip and lumbar spine were nearly equal throughout the DS phase.
3. Rotational movement of the hips played a more significant role during the DS phase than during the BS phase, which suggested that hip rotation was quite efficient during the DS phase.

This study was designed to provide basic information in order to investigate golf swing-specific factors associated with LBP. Future researches should involve analysis of golfers with a history of previous lumbar spine injury, and an in-depth understanding will need to be obtained by comparing such golfers with a control group. Additionally, electromyography data of muscle groups around hip and lumbar spine among golfers with LBP allow to develop the effective rehabilitation and therapeutic strategies.
